# Exploring the potential confounder of nitrogen fertilizers in the relationship between pesticide exposures and risk of leukemia: a Poisson regression with two-way fixed-effects analysis

**DOI:** 10.1186/s40880-017-0225-4

**Published:** 2017-07-14

**Authors:** Keith Fluegge, Kyle Fluegge

**Affiliations:** 1 Institute of Health and Environmental Research, Cleveland, OH 44118 USA; 2 New York City Department of Health and Mental Hygiene, New York, NY 11101-4132 USA

## Dear Editor,

Research discussing potential environmental toxins that may be related to the etiology of childhood leukemia has been growing. The suspected environmental contaminants include solvents, air pollutants, pesticides, and tobacco smoke. Exposure to various pesticides has come under particular scrutiny, with positive associations with childhood leukemia [1]. Poynter et al. [2] conducted a population-based study assessing the association between self-reported chemical exposures and odds of acute myeloid leukemia (AML) and myelodysplastic syndromes (MDS). The authors found no clear association between pesticides and AML or MDS; however, they did report significant associations of AML and MDS with other chemicals, including benzene, vinyl chloride, and fertilizers [2]. While it is certainly important to identify exposure to pesticides as a potential etiological factor in leukemia onset, it is also important to address confounding variables, such as fertilizers, that may be both strongly associated with pesticide use and empirically associated with leukemic outcomes.

We have previously identified environmental exposure to nitrous oxide (N_2_O), an agricultural and combustion pollutant, as a likely effect modifier to the proposed relationship between the use of the herbicide, glyphosate, and neurodevelopmental outcomes like attention-deficit hyperactivity disorder (ADHD) [3]. We found that use of glyphosate was closely tied to the use of nitrogen fertilizers in agriculture at a county urbanization level [3]. Therefore, it is possible that pesticide exposures may act as a proxy for air pollutants (i.e., N_2_O) directly related to the use of anthropogenic nitrogen in agriculture. Prior studies have identified pre-morbid ADHD and other developmental abnormality in children newly diagnosed with leukemia [4]. Therefore, if environmental N_2_O is a trigger for neurodevelopmental disorders, as we have suggested, and developmental abnormalities may precede childhood leukemia, we hypothesize that chronic exposure to N_2_O in the environment, and not necessarily pesticide exposures, may foster both neurodevelopmental and hematologic abnormalities.

To investigate the possible association between farm use of nitrogen fertilizers—as the most relevant environmental proxy for N_2_O emissions—and hospitalization for blood-related cancers, we have replicated our earlier work [3] using the database from the Healthcare Cost and Utilization Project (HCUP). We conducted a Poisson regression analysis using a two-way fixed-effects model. This approach minimizes the likelihood of omitted variable bias due to unobserved or unmeasured factors that influence the outcome. Briefly, a random variable *Y* is said to have a Poisson distribution with parameter *μ* if it takes integer values *y* = 0, 1, 2, … with probability.

$$Pr\left\{ {Y = y} \right\} = \frac{{e^{ - \mu } \mu^{y} }}{y!}$$

for *μ* > 0. The mean and variance of this distribution can be shown to be E(*Y*) = var(*Y*) = *μ*. We have a sample of *n* observations of discharges related to blood-related cancers, *y*_1_, *y*_2_,…, *y*_*n*_, which are treated as realizations of independent Poisson random variables, with *Y*_ij_, ~*P*(*μ*_ij_), where *i* represents a state and *j* an observation year. We let the logarithm of the mean depend on a vector of time-varying explanatory variables, *x*_ij_, such that the log-linear model is the following: $\log (\mu_{ij} ) = x_{ij}^{\prime} \beta_{1}$. We have a multiplicative model for the mean discharges by exponentiation: $\mu_{ij} = \exp \{ x_{ij}^{\prime} \beta_{1} \}$. In each case, the exponentiated regression coefficient exp{*β*_1ijk_} yields an incidence rate ratio (IRR), which represents a multiplicative effect of the *k*th predictor on the mean. A one log-unit increase in *x*_*k*_ multiplies the mean by a factor exp{*β*_1k_}.

Table 1 shows the results of our Poisson regression analysis using a two-way fixed-effects estimator. The unadjusted model indicates a reduced IRR for hospitalization for leukemia for every one log-unit increase in farm use of nitrogen fertilizers. No other blood cancer (non-Hodgkin’s lymphoma or multiple myeloma) was significant in the unadjusted models. When accounting for non-farm use of nitrogen fertilizers as well as the use of pesticides, including atrazine, cypermethrin, dicamba, 2,4-D, glyphosate, and 2-methyl-4-chlorophenoxyacetic acid (MCPA), a one log-unit increase in farm use of nitrogen fertilizers was protective against hospitalization for all three blood cancers. Data for many agricultural states in the USA (such as Iowa, Kansas, and Minnesota) were not available. A third adjusted model excluding the relatively rarely used pesticides, cypermethrin and MCPA, confirms the initial unadjusted model, suggesting that the extreme variability in these two pesticides, in particular, may have contributed to spurious results. However, the reduced risk of hospitalization for leukemia remained statistically significant for all models, lessening the likelihood this result can be attributed to chance. Similar results were noted when using a specific diagnosis of AML in the International Classification of Disease, 9th Revision–Clinical Modification (ICD-9-CM) as the dependent variable.

**Table 1:** Association between 1-year lagged nitrogen use and cancer outcomes

Outcome	CCS^a^	Model 1^b^		Model 2^c^		Model 3^d^	
		IRR (*n* = 249)	95% CI	IRR (*n* = 90)	95% CI	IRR (*n* = 249)	95% CI
Leukemia	39	0.95	0.93–0.98^*^	0.93	0.88–0.98^*^	0.95	0.93–0.98^*^
NHL	38	0.98	0.94–1.02	0.92	0.87–0.98^*^	0.97	0.94–1.01
MM	40	0.99	0.94–1.03	0.92	0.86–0.99^*^	0.98	0.94–1.03

*IRR* incidence rate ratio, *CI* confidence interval

^*^ *P* < 0.05; residual deviance exceeds degrees of freedom so robust standard errors were applied using *R* (packages ggplot2 and sandwich). The fixed-effects approach demands a great number of parameter estimates which did not properly converge in our negative binomial regression, creating the incidental parameter bias and invalidating this option to correct for model overdispersion. The conditional and unconditional Poisson fixed-effects analyses produce identical estimates, however

^a^Using all International Classification of Diseases, Ninth Revision, Clinical Modification (ICD-9-CM) codes included in the Clinical Classification Software (CCS) in HCUP (1997–2007), as described in our prior investigation [3]

^b^Unadjusted model, farm use of nitrogen fertilizers, as described in our prior investigation [3]

^c^Controlling for a 1-year lagged, logged measures of non-farm use of nitrogen fertilizers and pesticides, including atrazine, cypermethrin, dicamba, glyphosate, 2,4-D, and 2-methyl-4-chlorophenoxyacetic acid (MCPA), that are associated with arable agriculture crop production (United States Geological Survey, USGS) high estimates, years: 1996–2006) [3]

^d^Removing cypermethrin and MCPA data due to missingness

Including an interaction term of time with farm use of fertilizer heightens the significance of the main effect considerably, while the interaction itself significantly increases annual hospitalization risk for leukemia by 12% (results not shown). These data suggest that a 1-year lagged indicator of farm use of nitrogen fertilizer protects against hospitalization for leukemia, but that compensatory biological mechanisms may be induced over the longer term, soon increasing risk of hospitalizations for leukemia, as supported by Poynter et al. [2].

Biological evidence indicates that N_2_O may be protective against leukemic cell growth via the role of N_2_O in oxidizing the cobalt ion within cobalamin, inactivating vitamin B12 [5]. Also, N_2_O exposure may increase leukocyte DNA damage in patients who underwent surgery, as evidenced by a two-fold increase in the percentage of DNA intensity in the comet tail using digital fluorescence microscopy [6]. The extent of DNA damage was also positively correlated with the duration of N_2_O exposure [6]. Therefore, the current epidemiological finding of a significantly protective effect of farm use of nitrogen fertilizers (an environmental proxy of N_2_O emissions and exposures) against hospitalization for leukemia is consistent with the prevailing biological evidence. It is interesting to note, however, that elevated blood cobalamin has been acknowledged as a diagnostic marker for leukemia [7], although the significance of this metabolic abnormality in blood cancers is not well characterized. Moreover, the use of methylphenidate, a psychostimulant used to manage premorbid neuropsychiatric conditions like ADHD, has been found to be associated with increased risk of leukemia [8], although evidence of cytogenetic damage attributed to the use of methylphenidate is not consistent [9], suggesting that factors related to the use of psychostimulants and neurodevelopmental impairment, including environmental N_2_O exposure, may increase risk of leukemia. These data point to both endogenous and pharmacologic compensatory mechanisms, including leukemic outcomes and methylphenidate use respectively, which may reverse the hematologic and neuropsychiatric effects of chronic N_2_O exposure.

The present longitudinal findings provide a more nuanced perspective regarding the significantly increased odds of AML from self-reported exposure to fertilizer [2]. We suggest here that leukemic outcomes, including elevated blood cobalamin levels, may reflect compensatory mechanisms to reverse the hematologic depression from chronic environmental exposure to N_2_O emissions associated with nitrogen fertilizer use [10]. Additional investigations are therefore warranted to better characterize the metabolic role of increased serum vitamin B12 in hematologic cancers. As confirmed by Poynter et al. [2], future investigations exploring links between pesticides and leukemia should consider the associated use of nitrogen fertilizers and chronic exposure to related air pollutants, such as N_2_O, which has been empirically shown to affect not only leukemic outcomes but also neurodevelopmental abnormality that may often precede a leukemia diagnosis. Therefore, we think that nitrogen fertilizers and their influence on nitrous oxide emissions should be considered.

## Abbreviations

ADHD: attention-deficit hyperactivity disorder
AML: acute myeloid leukemia
CCS: Clinical Classification Software
HCUP: Healthcare Cost and Utilization Project
IRR: incidence rate ratio
ICD-9-CM: International Classification of Disease, 9th Revision–Clinical Modification
MDS: myelodysplastic syndromes
MM: multiple myeloma
N_2_O: nitrous oxide
NHL: non-Hodgkin’s lymphoma

## References

[1] Bailey HD, Infante-Rivard C, Metayer C, Clavel J, Lightfoot T, Kaatsch P (2015). Home pesticide exposures and risk of childhood leukemia: findings from the Childhood Leukemia International Consortium. Int J Cancer.

[2] Poynter JN, Richardson M, Roesler M, Blair CK, Hirsch B, Nguyen P (2017). Chemical exposures and risk of acute myeloid leukemia and myelodysplastic syndromes in a population-based study. Int J Cancer.

[3] Fluegge K, Fluegge K (2017). Exposure to ambient PM10 and nitrogen dioxide and ADHD risk: a reply to Min and Min (2017). Environ Int.

[4] Janzen LA, David D, Walker D, Hitzler J, Zupanec S, Jones H (2015). Pre-morbid developmental vulnerabilities in children with newly diagnosed acute lymphoblastic leukemia (ALL). Pediatr Blood Cancer.

[5] Abels J, Kroes AC, Ermens AA, van Kapel J, Schoester M, Spijkers LJ (1990). Anti-leukemic potential of methyl-cobalamin inactivation by nitrous oxide. Am J Hematol.

[6] Chen Y, Liu X, Cheng CH, Gin T, Leslie K, Myles P (2013). Leukocyte DNA damage and wound infection after nitrous oxide administration: a randomized controlled trial. Anesthesiology.

[7] Ermens AA, Vlasveld LT, Lindemans J (2003). Significance of elevated cobalamin (vitamin B12) levels in blood. Clin Biochem.

[8] Oestreicher N, Friedman GD, Jiang SF, Chan J, Quesenberry C, Habel LA (2007). Methylphenidate use in children and risk of cancer at 18 sites: results of surveillance analyses. Pharmacoepidemiol Drug Saf.

[9] Witt KL, Shelby MD, Itchon-Ramos N, Faircloth M, Kissling GE, Chrisman AK (2008). Methylphenidate and amphetamine do not induce cytogenetic damage in lymphocytes of children with ADHD. J Am Acad Child Adolesc Psychiatry.

[10] Kano Y, Sakamoto S, Sakuraya K, Kubota T, Kasahara T, Hida K (1983). Effects of nitrous oxide on human cell lines. Cancer Res.

